# Infertile Couples Prefer Twins: Analysis of Their Reasons and Clinical Characteristics Related to This Preference

**Published:** 2018

**Authors:** Rosario Mendoza, Teresa Jáuregui, Maria Diaz-Nuñez, Mariana de la Sota, Alaitz Hidalgo, Marcos Ferrando, Lorea Martínez-Indart, Antonia Expósito, Roberto Matorras

**Affiliations:** 1- Human Reproduction Unit, Cruces University Hospital, Bizkaia, Spain; 2- Centro de Psicología Mariana de la Sota, Bizkaia, Spain; 3- IVI Bilbao, Bizkaia, Spain; 4- Clinical Epidemiology Unit, Cruces University Hospital, Bizkaia, Spain

**Keywords:** IVF, Questionnaire, Twins

## Abstract

**Background:**

The number of multiple pregnancies has been significantly increased in the last decades due to assisted reproduction techniques development. Compared to singleton, twins and multiple pregnancies are associated to more complications and risks for both mother and children. The objective of this study was to examine the proportion of patients preferring a multiple birth over a singleton after an IVF/ICSI attempt, their reasons and the influence of socio-demographic and clinical parameters on their preference.

**Methods:**

A prospective study was conducted in two different Spanish centers in 2014; a public university hospital and a private clinic, with different populations and embryo transfer policies. In order to evaluate patients and partners attitudes towards twins and singletons, an anonymous 10-question survey was conducted and 399 were invited to participate.

**Results:**

58.2% of participants preferred having twins to having one child at a time and 4.8% preferred triplets. Primary reasons for preferring twins were “avoiding a new IVF/ICSI attempt” (61.6%), “I like the idea of having twins” (27.3%), “avoiding the waiting list” (5.8%), and “in my opinion with the latest technology, the rate and severity of complications in multiple pregnancies are low” (5.2%). The multivariate analysis showed that the only significant parameter related to a preference for multiplets was the transfer of women’s own fresh embryos (OR=3.31).

**Conclusion:**

Twin pregnancy risks are not perceived as important by the majority of IVF/ICSI couples, and many of them specifically prefer twins. In our opinion, much more information is needed highlighting the multiple pregnancy risks and that information should come from medical sources besides general media.

## Introduction

In recent decades, as a consequence of assisted reproductive technology (ART), there has been a dramatic increase in multiple pregnancies (1, 2). Such pregnancies are associated with a higher rate of complications for both the mother and the infant (3), and also with a number of social, psychological, and economic consequences (4). Given this, multiple pregnancies in general and twins in particular are considered an adverse/unwanted effect of ART by specialists in reproductive medicine. It has been suggested that multiple births could be better accepted in the Mediterranean societies due to tradition of large families. In Spain, Assisted Reproduction law has been observed since 2006 specifically forbidding transfers of more than 3 embryos (5). Hence, a number of strategies have been designed to reduce rates of twin/multiple pregnancies associated with ART (6). A number of authors have stressed the importance of single embryo transfer (SET) in IVF/ICSI cycles as the only way to eradicate twin pregnancies (7–10). The Spanish fertility Society also recommends single embryo transfer in good prognosis cases (11). However, whereas there is little doubt that from perinatal and obstetric points of view, single pregnancies are preferable, it is not clear whether infertile couples prefer singletons or twins (12–14). Notably, some authors report a high proportion of infertile couples preferring twins over singletons (15–17). Under such circumstances, the efforts of medical staff to reduce twin pregnancy rates would not have much impact if the desire of patients is having twins (18).

The aims of our work were to analyze the preferences of IVF/ICSI couples regarding multiplicity in two different Spanish centers (one public and one private), and to study the influence of socio-demographic and clinical characteristics on the aforementioned preferences.

## Methods

A prospective study was undertaken giving a survey questionnaire containing 10 questions to all women (n=399) and their male partners (n= 399) attending two ART centers 1 *hr* before embryo transfer after an IVF/ICSI cycle (between February 2014 and August 2014).

These centers were public providers, the Reproductive Unit at Cruces University Hospital (n= 576) and a private fertility clinic, Instituto Valenciano de Infertilidad (IVI) (n=222) in Bilbao, the Basque Country, Spain. The study was approved by our Institutional Ethical Board (code CEIC 14/35). Informed consent was obtained the day where the questionnaire was given. At the first consultation, all the couples undergoing IVF/ICSI had received verbal and written information from their gynecologist regarding the IVF/ICSI procedures, including related risks, especially multiple pregnancy.

A 10 item questionnaire designed by our group (Annex 1) was used to evaluate patients’ opinion concerning multiplicity and IVF. The questionnaire was previously validated twice in two different subgroups of infertile patients, and some adjustments were made. The couples for whom the validation was performed were not included in the present study.

The woman and the man were asked to fill out the questionnaire independently while waiting for embryo transfer, before any clinical counseling about the number of embryos to be transferred. The questionnaire was collected 30 *min* later, in a closed envelope. In total, 399 women and 399 men correctly completed the questionnaire. Single women were not included in the study. No individuals declined to complete the questionnaire.

The participation in the study was offered to all the couples fulfilling the inclusion criteria during the study period. The acceptance rate was 100%. Since a number of different parameters were considered and in a number of them, there were no previous data, sample size estimation was not done beforehand.

The populations and embryo policies were different in the two centers. In patients from the public hospital (n=576), all the transfers corresponded to their own fresh embryos, whereas in patients from the private clinic (n=222), 66 patients had their own fresh embryo transfers, 55 patients had their own cryopreserved-thawed embryo transfers, 75 patients received embryos from donor oocytes (fresh or vitrified), and the other 26 patients did not answer the corresponding question.

Concerning embryo policy during the study period, at the public hospital, the policy was to recommend transfer of two embryos in a first or second attempt in women under 37 years of age with at least two good quality embryos, while in women under 30, only one embryo was transferred, and three embryos were transferred in poor prognosis cases. Overall, the percentages of single, double and triple embryo transfers were 17.7, 46.6, and 35.7%, respectively. At the private clinic, the policy was to recommend transfer of one embryo in good prognosis cases; women under 35 years of age, and two embryos in all other cases. The percentages of single and double embryo transfer were 28% and 72%, respectively.

In the public hospital, the waiting list for an IVF/ICSI cycle was 10–12 months, whereas in the private clinic there was no waiting list. On the other hand, in the public hospital, the whole IVF cycle and the drugs were completely free of charge for patients, whereas in the private clinic, patients received no reimbursements and had to pay for the complete cost of IVF treatment and drugs. In the public hospital, three IVF cycles were offered to women under 40 years of age, and low ovarian reserve (19) was an exclusion criterion. In the private clinic, no fixed limits were set concerning the number of cycles, age of the woman, or ovarian reserve.

Separate copies of the questionnaire were given to each partner of the couple, and though questionnaires were anonymous, a code enabled us to match the responses of the men and women from each couple.

Data were analyzed using the IBM SPSS Statistics for Windows, Version 21. Qualitative variables were studied with Chi-squared or Fisher’s exact tests. The degree of agreement above chance was assessed with the kappa statistic (Landis and Koch, 1977) (20). The influence of variables on the desire for multiple pregnancy was analyzed by univariate and multivariate logistic regression. Results were expressed using odds ratios (ORs) and corresponding 95% confidence intervals (CIs). The p<0.05 was considered statistically significant.

## Results

### Demographic and clinical characteristics:

Mean age of women was 35.9±3.4 years (mean±SD), and mean age of men 38.0±4.5. Mean duration of infertility was 4.5±2.4 and 85.3% of women were nulliparous. The number of previous children was 0.18±0.47. The mean number of previous IVF/ICSI cycles was 1.18±1.2 (67.3% of women had a previous cycle). The proportion of primary education was 15.7% in women and 23.1% in men; the proportion of secondary education was 32.9 and 40.2%, whereas university degree represented 51.4 and 36.7%. Also, 13.6% of professions related to health care; 3.5% nursing, 1.1% medicine, 10.8% others (pharmacy, biology, ...).

A total of 798 questionnaires were analyzed. With respect to the main question of the survey, “How many children would you like to have as the result of this embryo transfer?”, 58.2% of patients preferred having twins to having one child at a time (37.1%), and 4.8% preferred triplets.

The most frequently cited primary reasons for preferring one child at a time were “lower risks for the mother” (37.4%) and “I want to have just one child in my life” (31.5%), followed by “economic considerations” (16.1%) and “lower risks for the baby” (12.2%). The main secondary reasons were “lower risks for the baby” (52.2%) and “lower risks for the mother” (28.4%) (Table 1).

**Table 1.** Reasons for preferring one child or twins at this transfer

**Primary reason for preferring one child:**	**%**	**Primary reason for preferring twins:**	**%**
“lower risk for the mother”	37.4	“avoiding a new IVF attempt”	61.6
“I want to have just one child in my life”	31.5	“I like the idea of twins”	27.3
“economic considerations”	16.1	“avoiding the waiting list”	5.8
“lower risks for the baby”	12.2	“in my opinion, the rate and severity of complications in multiple pregnancies are low”	5.2
**Secondary reason for preferring one child:**		**Secondary reasons for preferring twins:**	
“lower risks for the baby”	52.2	“I like the idea of twins”	39.6
		“avoiding a new IVF attempt”	27.2
“lower risks for the mother”	28.4	“avoiding the waiting list”	16.6
		“in my opinion, the rate and severity of complications in multiple pregnancies are low”	16.6

On the other hand, the primary reason most often cited for preferring twins was “avoiding a new IVF attempt” (61.6%), followed by “I like the idea of having twins” (27.3%), “avoiding the waiting list” (5.8%), and “in my opinion, the rate and severity of complications in multiple pregnancies are low” (5.2%). Economic considerations were reported by 16.1%. Secondary reasons cited were “I like the idea of having twins” (39.6%), “avoiding a new IVF attempt” (27.2%), “avoiding the waiting list” (16.6%), and “in my opinion, the rate and severity of complications in multiple pregnancies are low” (16.6%) (Table 1). If data regarding twins and triplets were combined, the results were similar.

Regarding the influence of socio-demographic characteristics on multiplicity preferences (Table 2), nulliparity was significantly associated with a higher preference for twins; 63.9% *vs*. 27.4% in couples when the woman or the man had at least one previous child (p<0.01). No differences were observed considering gender, previous IVF attempts, level of education, or occupation category. When the occupation was related to medicine, there was a trend in having less desire for twins (25.0%), but a stronger desire for triplets (12.5%), though these differences were not statistically significant (Table 2).

**Table 2.** Demographic factors and number of children desired in this cycle

	**One (%)**	**Two (%)**	**Three (%)**	**p**
**Number of desired children**	37.1	58.2	4.8	
**Women**	35.8	59.6	4.5	
**Men**	38.3	56.7	5.0	ns
**Previous IVF/ICSI cycles**				
No	37.3	59.2	3.5	ns
Yes	36.2	58.6	5.2	
**Previous children**				
No	31.4	63.9	4.7	
Yes	69.2	27.4	3.4	<0.01
**Study level**								
Primary	36.8	58.1	5.2	
Graduate	39.0	55.8	5.1	ns
University	35.7	59.9	4.3	
**Profession**				
Not related to healthcare	36.2	58.9	4.9	
**Related to healthcare**				ns
Nurse	32.1	60.7	7.1	
Medicine	62.5	25.0	12.5	
Others(Pharmacy, Biology, etc)	43.0	54.7	2.3	

Regarding the clinical setting, significant differences were found between the reproductive centers; 62.2% of patients from the public hospital preferred twins, and 5.6% preferred triplets *vs.* 47.7% and 2.7% of patients from the private clinic (p<0.01) (Table 3). However, in the public hospital, all the transfers corresponded to their own fresh embryos, and when the analysis was restricted to this type of transfer, the preference for twins was similar in the two centers (62.2% and 60.6%). Among patients receiving donor oocytes (fresh or cryopreserved), the rate of preferring twins was also similar (54.6%). The preference for twins was only markedly lower (36.4%) among those receiving cryopreserved-thawed embryo transfers (n= 55). This different pattern was related to a relatively high proportion of those receiving cryopreserved-thawed embryos having at least one previous child (36.4%, n=20), 90% of this subgroup preferring a singleton. Indeed, among patients receiving cryopreserved-thawed embryo transfers who did not have any previous children (63.6%, n=35), the preference for twins was closer to that in other groups (51.4%).

**Table 3.** Preference for multiple births by center

	**Public hospital (%)**	**Private clinic (%)**	**p**
**Preference for twins**	62.2	47.7	<0.001
**Preference for triplets**	5.6	2.7	
**Preference for twins**			
Own fresh embryos	62.2	60.6	
Donor oocytes (fresh or vitrified)	-	54.6	
Frozen-thawed embryos	-	36.4	

When the logistic regression analysis was performed, univariate analysis indicated the following factors were significantly associated with wanting multiple births; age (OR=1.39; CI 1.04–1.21), infertility duration (OR=1.13; CI=1.05–1.21), no previous children (OR=4.91; CI=3.21–7.52), public hospital (OR=2.06; CI=1.5–2.82), and their own fresh embryo transfers (OR=3.31; CI=1.89–5.89). However, when multivariate analysis was performed to identify independent risk factors, the only significant factor was the use of own fresh embryos for transfers (OR=3.31; CI= 1.89–5.89).

A moderate agreement was found in the desire for multiple births between the members of each couple considering the scale of Landis and Koch (1977) (16) (kappa=0.601) (data not shown). Concerning the reasons cited for their singleton/multiple preferences, there was also a moderate agreement (kappa=0.534) (data not shown).

## Discussion

In our study, almost 60% of our infertile couples specifically preferred twins. It has to be highlighted that this had nothing to do with a preference concerning receiving a two- embryo transfer, where pregnancy rate could be increased and twin pregnancy was accepted as a related adverse effect. Our couples specifically wanted to have twins, by a number of different reasons.

Obstetricians/gynecologists and neonatologists are acutely aware of the risks inherent in pregnancies and deliveries involving multiple fetuses, as well as the increased morbidity and mortality among infants born from multifetal pregnancies (13, 21). Moreover, the economic effect on society and the economic and emotional stress on families associated with raising twins, triplets, and more children are becoming increasingly apparent (13). In this context, a number of scientific associations promote SET (10, 11, 21, 22), at least in good prognosis cases, with multiple pregnancy, even twins, and consider adverse effects or at least an unwanted effect of ART. However, the same may happen in ART when facing a similar phenomenon called “automobile industry paradox” (many drivers wanting vehicles that run much faster than the recommended/legal speed limits) (23) in which infertile patients specifically want multiple births.

In Europe, four family models have been described; Anglo-Saxon, Continental, Nordic and Mediterranean (17). It has been suggested that multiple births could be better accepted in the Mediterranean societies due to a tradition of large families. In fact, in European Society of Human Reproduction and Embryology registers, the highest rates of three embryo transfers are usually found in Mediterranean countries (24). In Spain, although there is a tradition of large families, in the last decades, there has been a dramatical reduction of the mean number of children per couple, mainly as a consequence of contraception availability and the delayed maternity, because of social, professional and economic reasons. In the last available data from the Spanish National Statistics Institute in 2016, the mean number of children per couple was 1.34. Similarly, the age of the woman at her first labor progressively increases, now being 32.0.

Based on our knowledge, the desire for multiple pregnancies has not been analyzed in the Mediterranean infertile population. Various studies have found infertile patients preferring twins from 20.3% in Iowa, USA (13), 21% in Chicago, USA (17), 32% in Aberdeen, UK (12), and 38.9% in Canada (16), 39.7% (women) and 42.1% (men) in Kuala Lumpur, Malaysia (25), to 58.7% (14) and 84.7% (15) in two different studies in Denmark. In Spain, rates of preferring multiple births were 63% overall, and 58.2% for twins, in the upper range of previously reported data. This result is observed despite our survey having been performed around a decade later in comparison to other studies and the fact that the greater current awareness among reproductive clinicians of the risks could have been expected to influence preferences.

In univariate analysis of the sociodemographic data, the following variables were found to be positively associated with wanting multiple births; the woman’s age, infertility duration, nulliparity, treatment in a public hospital and transfers of women’s own oocyte fresh embryo. Age was found to be associated with wanting twins in one previous report (16), but not in others (14, 15). In our univariate analysis, wanting a multiple birth was not associated with gender, the number of previous IVF/ICSI attempts, and level of educational or occupation category.

In our multivariate analysis, however, the transfer of cryopreserved-thawed embryos was the only variable associated with wanting multiple pregnancies (36.4%). While in a previous multivariate analysis it was shown that lower family income was independently associated with the desire for multiple births (13), in our study neither educational level nor occupational status were associated with the preference for multiple births, probably due to the fact that in our public hospital, IVF/ICSI cycles are offered free of charge.

It should be highlighted that even in couples desiring singletons, the risk for the infant did not seem to weigh heavily in the decision-making process, it being the main reason for desiring singletons in only 12.2% of our sample. The “lower risk for the mother” was considered a much more relevant factor, the most important, accounting for 37.4% of cases, somewhat higher than ideological reasons (“I want to have just one child in my life”, 31.5%). A similar pattern was observed in couples preferring multiple births; “in my opinion, the rate and severity of complications in multiple pregnancies are low” was cited as the main reason in 5.2% of cases and again ideological reasons were common (“I like the idea of twins”, 27.3%), but the most important reason, by far, was “avoiding a new IVF attempt”, cited by 61% of participants. Notably, in couples from the public hospital, “avoiding the waiting list” was only cited as the most important reason in 7.2% of cases. From our data, it is impossible to ascertain whether couples chose “avoiding a new IVF attempt” due to a desire for an instant family (16) or to avoid the stress of a further IVF cycle. The financial cost of a new IVF attempt does not seem to be an important reason, in the public hospital (where no charges are levied for the cycle or the drugs required). Also, well over half of couples (60.8%) selected “avoiding a new IVF attempt” as the main reason for preferring twins.

It has been reported that, in IVF, men place more importance on side effects while women highlight success rate (18). In a previous study, the preference for twins was similar in men and women, but no matched analysis within each couple was performed (16). It was found that the positioning regarding multiple births was very similar in men and women and the agreement coefficient was moderate (kappa=0.601). Further, a moderate agreement (kappa=0.534) was observed between the reasons cited for desiring singleton or multiple births by each member of the couple. In our opinion, this reflects the heavy weight of ideological reasons, and it is plausible to suppose that they would be similar within each couple.

It should be highlighted that our study did not focus on preferences for SET/DET. That is, couples were not asked whether they accepted the risk of a twin pregnancy, a potential outcome in DET. In this context, 58% specifically wanted a twin birth, which is (almost) incompatible with SET. On the other hand, it should be stressed that the rates of wanting twins were almost identical considering only cycles with women’s own fresh embryos, despite different embryo transfer policies in the two centers (SET being performed in 28% of cases and there being no triple embryo transfers in the private clinic vs 18% SET and 36% triple embryo transfers in the public hospital). The remaining embryo origin categories could not be compared, since they were not an option in the public hospital.

For the moment, it can be concluded that SET is difficult to implement in our country, since twin pregnancy risks are not perceived as important by the majority of couples involved, and many, irrespective of their sociodemographic characteristics, specifically prefer twins. It is not clear whether additional information (21, 25) would change patient attitudes towards twin pregnancies. In agreement with this, in a very recent work made in another Spanish center, nearly half of the patients refused elective SET even after having been well informed of its benefits (26).

## Conclusion

More studies would be needed to assess if specific multiple pregnancy information programs could change patients’ preferences. However, it should be highlighted that the main reason for preferring twins was “avoiding a new IVF attempt”. Thus, perhaps making new IVF attempts less cumbersome, reducing prices in private centers, reducing waiting lists in public centers, and developing patient-friendly IVF protocols would be able to reduce the fear of repeating cycles and, in turn, reducing the dominant preference for twins.
